# Effects of Chronic Administration of Clenbuterol on Contractile Properties and Calcium Homeostasis in Rat Extensor Digitorum Longus Muscle

**DOI:** 10.1371/journal.pone.0100281

**Published:** 2014-06-27

**Authors:** Pascal Sirvent, Aymerick Douillard, Olivier Galbes, Christelle Ramonatxo, Guillaume Py, Robin Candau, Alain Lacampagne

**Affiliations:** 1 Clermont Université, Université Blaise Pascal, EA 3533, Laboratoire des Adaptations Métaboliques à l'Exercice en Conditions Physiologiques et Pathologiques (AME2P), BP 80026, Aubière, France; 2 U1046, INSERM, Université Montpellier 1, Université Montpellier 2, 34295 Montpellier, France; 3 CHRU Montpellier, 34295 Montpellier, France; 4 National Institute for Agronomic Research (INRA), UMR 866 Muscular Dynamic and Metabolism, University of Montpellier, Montpellier, France; University of Debrecen, Hungary

## Abstract

Clenbuterol, a β_2_-agonist, induces skeletal muscle hypertrophy and a shift from slow-oxidative to fast-glycolytic muscle fiber type profile. However, the cellular mechanisms of the effects of chronic clenbuterol administration on skeletal muscle are not completely understood. As the intracellular Ca^2+^ concentration must be finely regulated in many cellular processes, the aim of this study was to investigate the effects of chronic clenbuterol treatment on force, fatigue, intracellular calcium (Ca^2+^) homeostasis and Ca^2+^-dependent proteolysis in fast-twitch skeletal muscles (the extensor digitorum longus, EDL, muscle), as they are more sensitive to clenbuterol-induced hypertrophy. Male Wistar rats were chronically treated with 4 mg.kg^−1^ clenbuterol or saline vehicle (controls) for 21 days. Confocal microscopy was used to evaluate sarcoplasmic reticulum Ca^2+^ load, Ca^2+^ -transient amplitude and Ca^2+^ spark properties. EDL muscles from clenbuterol-treated animals displayed hypertrophy, a shift from slow to fast fiber type profile and increased absolute force, while the relative force remained unchanged and resistance to fatigue decreased compared to control muscles from rats treated with saline vehicle. Compared to control animals, clenbuterol treatment decreased Ca^2+^-transient amplitude, Ca^2+^ spark amplitude and frequency and the sarcoplasmic reticulum Ca^2+^ load was markedly reduced. Conversely, calpain activity was increased by clenbuterol chronic treatment. These results indicate that chronic treatment with clenbuterol impairs Ca^2+^ homeostasis and this could contribute to the remodeling and functional impairment of fast-twitch skeletal muscle.

## Introduction

Clenbuterol is a β_2_-selective adrenergic receptor agonist primarily used for treating asthma. Like other β–adrenoceptor agonists, clenbuterol can induce skeletal muscle hypertrophy, by stimulating protein synthesis and inhibiting proteolysis [1], [2]. Therefore, many studies have focused on the potential use of clenbuterol and some other β–adrenoceptor agonists to counteract muscle wasting associated with many pathological conditions [3]. Although clenbuterol has positive effects on skeletal muscle mass, some studies also reported myotoxic effects on heart and skeletal muscles [4], [5]. Moreover, it is well documented that administration of β–adrenoceptor agonists, such as clenbuterol, results in a dramatic shift from a slow-oxidative to fast-glycolytic muscle fiber type profile [6], [7], [8]. This change in muscle fiber composition increases muscle fatigue, as reported after chronic administration of β–adrenoceptor agonists [9].

Clenbuterol effects on skeletal muscle seem to be fiber-type specific. Indeed, clenbuterol treatment causes hypertrophy only of fast skeletal muscle fibers [10]. Conversely, its myotoxic effects selectively target slow fibers [4], [5], [11], [12].

The cellular mechanisms explaining the effects of chronic β–adrenoceptor agonist administration on skeletal muscle are not completely understood. Regulation of intracellular Ca^2+^ concentration is implicated in many cellular processes, such as excitation-contraction coupling, cell survival, energetic metabolism and gene transcription signaling [13]. Following depolarization, the Ca^2+^ transient, which is mainly elicited by the Ca^2+^ release from Ryanodine receptors (RyR) and is strongly influenced by sarcoplasmic reticulum (SR) Ca^2+^ load, is a major determinant of skeletal muscle functional properties. Acute administration of clenbuterol on skinned skeletal muscle fibers has a direct effect on sarcoplasmic reticulum (SR) Ca^2+^ release [14], [15]. In cardiac muscle, chronic administration of clenbuterol increases SR Ca^2+^ content and the Ca^2+^-transient amplitude [16]. However, detailed studies on the impact of chronic clenbuterol treatment on calcium (Ca^2+^) homeostasis in skeletal muscle are lacking. Therefore, in this study, we assessed the effect on muscle Ca^2+^ homeostasis of chronic treatment (21 days) with clenbuterol in male Wistar rats. Real time laser scanning confocal microscopy was used to investigate Ca^2+^-transient amplitude, Ca^2+^ spark spatio-temporal properties, which reflect RyR functional properties, and SR Ca^2+^ load in EDL muscles. In addition, we also evaluated the effects of clenbuterol treatment on Ca^2+^-activated proteolysis because it could be altered by Ca^2+^ homeostasis disturbances. EDL muscles have a fast-glycolytic phenotype and were chosen because they are less affected by myonecrosis and they are more sensitive to clenbuterol-induced hypertrophy [4], [10]. Moreover, while the acute inhibitory effects of clenbuterol on force and Ca^2+^ transients in rat slow-twitch soleus muscle have been already described [15], its effects on fast-twitch skeletal muscles are less known.

## Materials and Methods

### Animals

This study was approved by the Ethics Committee on Animal Experimentation of the Languedoc Roussillon Region, France, in accordance with the guidelines from the French National Research Council for the Care and Use of Laboratory Animals (Permit Number: CEEA-LR-1069). Surgery was performed under sodium pentobarbital anesthesia and all efforts were made to minimize suffering. Twenty-two 6-weeks/old Wistar male rats (body mass: 236.3±3.8 g) were randomly allocated to the control (CTR; n = 11 animals) or clenbuterol-treated (CBL; n = 11) group. They were housed in standard cages with a 12 h∶12 h light-dark cycle and provided with food and water ad libitum. Every day at 9 am for 21 days, rats were weighted and received a subcutaneous injection of clenbuterol (4 mg.kg^−1^.day^−1^ diluted in saline solution) or of an identical volume of saline vehicle (CTR). Rats were sacrificed with a lethal dose of pentobarbital sodium 24 h after the last injection.

### Muscle preparation

For muscle contraction studies and Ca^2+^-signaling measurements, intact EDL muscles were surgically dissected and immediately placed in a custom-built Plexiglas chamber filled with Tyrode solution (121 mM NaCl, 5.0 mM KCl, 1.8 mM CaCl_2_, 0.5 mM MgCl_2_, 0.4 mM NaH_2_PO_4_, 24 mM NaHCO_3_, 0.1 mM EDTA 0.1, 5.5 mM glucose) equilibrated with 95% O_2_–5% CO_2_ gas and maintained at 25°C. The pH was set to 7.4.These conditions are optimal for maintaining muscle viability *in vitro* for the duration of the experimental protocol.

For calpain activity measurements, EDL muscles were homogenized in ice-cold buffer (20 mM Tris–HCl, 1.5 mM EDTA, 5 mM EGTA, 1 mM DTT 1, 0.15 nM Pefabloc, 5.8 nM Pepstatin A) and then centrifuged (1000*g* for 10 min, 4°C). Supernatants were stored at −80°C. The protein content of supernatants was determined using the bicinchoninic acid (BCA) protein assay (Pierce, Interchim, Montluçon, France).

For western blotting, muscles were homogenized in ice-cold lysis buffer (20 mM Tris, 100 mM NaCl, 1 mM EGTA, 20 mM NaF, 5% Triton X-100, 0.15 nM Pefabloc, 5.8 nM Pepstatin A) and then centrifuged (12 000*g* for 20 min, 4°C). Supernatants were stored at −80°C.

### Muscle phenotype and cross-sectional areas

Muscle phenotype was determined on cryosections (6 µm) by myosin ATPase histochemical staining as previously described [11]. The muscle-fiber cross-sectional area (CSA) was determined using the ImageJ analysis software and is expressed in µm^2^.

### Muscle fiber contractile function

EDL muscle fiber contractile properties were assessed *in vitro* according to the methods described in detail previously [7]. After 15-min equilibration in the bath, muscles were connected to a force transducer/length servomotor system (model 305B, Cambridge Instruments, Aurora Scientific Inc, Ontario, Canada) and were stimulated along their entire length with platinum wire electrodes. The optimum muscle length (L_0_), i.e. the muscle length producing maximal twitch tension, was determined. All subsequent measurements were made at L_0_. The tension-frequency response was then determined (701B Stimulator, Aurora Scientific Inc, Ontario, Canada) using stimulation trains of 500-ms, with pulse duration of 0.5 ms at frequencies of 1–100 hz. Stimulus trains were separated by one-min interval. The maximum isometric tetanic tension (P_0_) was then determined. Three minutes after the tension-frequency determination, the resistance to fatigue was evaluated using a low-frequency fatigue protocol of 50-hz trains of 700 ms delivered every 2 s for 5 min [17]. The muscle fatigue index (T_lim_) was defined as the time taken to produce a 50% reduction from the initial maximum power output. After all measurements, muscles were removed from the bath, trimmed of the connective tissue, blotted dry and weighed on an analytical scale. The specific force (sP_0_, expressed in N/cm^2^) was calculated by dividing the tension (P_0_) by the whole-muscle CSA. The whole-muscle CSA was determined by dividing the muscle volume (obtained by dividing the muscle mass by the muscle density, assumed to be 1.06 g/cm^3^) by the average fiber length (L_f_). [18]. L_f_ was determined by multiplying L_0_ by 0.44, which is the previously determined fiber length to muscle length ratio for the EDL muscle [7].

### Ca^2+^-transient measurements

Intact EDL muscles were loaded with 50 µM Rhod-2 AM (Molecular Probes, Eugene, OR), a fluorescent probe for intracellular Ca^2+^, in Krebs-Ringer solution for 1 h. Muscles were then mounted under stretch (between 3.1 and 3.4 µm, approximately the sarcomere length) to suppress movement artifacts [19]. Action-potentials and their associated Ca^2+^ transients were triggered by field stimulation with a single 2 ms pulse just above the stimulation threshold for visible fiber contraction under a microscope. Fluorescence images were acquired in line-scan mode (spatial [x] vs. temporal [t], 1.5 ms/line) with a Zeiss (Le Pecq, France) LSM 510 NLO confocal system (25× oil immersion, NA = 0.8), as previously reported [19]. Multiphoton excitation was obtained by using a 5-W Chameleon pulsed TI/sapphire laser (Coherent, Orsay, France) tuned at 835 nm. The emitted fluorescence was recorded at ∼560 nm. Single-fiber Ca^2+^-transients were identified on raw fluorescence microscopy images. Image strips of the manually identified area of a single fiber were extracted. After subtracting the photomultiplier tube (PMT) offset current, strip images were converted to ΔF/F, where F is the fiber mean resting fluorescence calculated from the image area preceding the action-potential stimulation and the subsequent Ca^2+^-transient. The temporal fluorescence time courses of each fiber were generated by spatial averaging of the ΔF/F images, resulting in the mean spatial F value for each temporal coordinate.

### Ca^2+^-spark measurements

Local Ca^2+^-release events (i.e., Ca^2+^-sparks) were measured as previously reported [20]. Muscle samples were immediately placed in an intracellular-like buffer (140 mM K-glutamate, 10 mM HEPES, 20 mM phosphocreatine, 5 mM Na_2_ATP, 4.53 mM MgCl_2_, 1 mM EGTA, 0.29 mM CaCl_2_, and 2 mM malate, pH 7.0). Single fibers were manually dissected under a dissecting microscope (Stemi 200; Zeiss, Le Peck, France), mounted in an experimental chamber containing the internal-like buffer, and permeabilized by adding 0.01% saponin at room temperature for 30 s. Fibers were slightly stretched to the sarcomere length (approximately 2.8–3.2 µm) to reduce movement artifacts. Fibers were bathed in internal-like buffer containing 50 µM of Fluo-3 (pentapotassium salt; Tef-labs, Austin, TX), a fluorescent Ca^2+^ indicator. Fluorescence images were acquired with the confocal system operated in line-scan mode (x vs. t, 1.5 ms/line). Fluo-3 was excited with an argon/krypton laser at 488 nm and fluorescence was recorded at 525 nm. Image acquisition was performed along the fiber longitudinal axis. Potential spark areas were empirically identified using an auto-detection algorithm [21]. The mean F value of the image was calculated by summing and averaging the temporal F at each spatial location, while ignoring potential spark areas. This F value was then used to create a ΔF/F image pixel by pixel. Following a three-point smoothing routine, potential spark locations were visualized manually and their spatio-temporal properties analyzed as described previously [19], [22]. We limited our analysis to Ca^2+^-spark activity corresponding to brief transients (<100 ms) of localized fluorescence. Determinations of the spatio-temporal properties of individual Ca^2+^-sparks were made on spatial (x) and temporal (t) profiles of sparks centered at the peak amplitude. Parameters derived from the ΔF/F temporal profile include its amplitude, the rise time and the time constant for the decay rate given by the time-to-half decay (Tlim). The width of the Ca^2+^-spark (full width at half maximum peak amplitude) was determined from the spatial profile. The time course and spatial width of the fluorescence were then assessed and the following criteria were then used to classify a perturbation of ΔF as a Ca^2+^-spark: Δ*F*/*F* amplitude >0.2, half duration >6 ms and <50 ms, and half width >0.7 µm and <4 µm.

### Evaluation of the sarcoplasmic reticulum Ca^2+^ content

The SR Ca^2+^ content was evaluated as previously described [23], [24]. Bundles of fibers were prepared as described for Ca^2+^-spark measurement, including permeabilization with saponin. Ca^2+^ release was elicited by adding to the 250 µl of internal-like buffer (containing the fluorescent Ca^2+^ indicator Fluo-3) in which the fibers were bathed another 250 µl of the same solution with 100 µM of 4-chloro-m-cresol (4-CmC). Ca^2+^-transient was imaged using fast time series of x–y confocal images (25× oil immersion, NA = 0.8; Zeiss). Fields of one or two fibers were imaged at the acquisition rate of one image every 400 ms. Fluo-3 was excited with an argon/krypton laser at 488 nm, and fluorescence was recorded at 525 nm. The mean fluorescence was calculated in regions of interest and reported as a function of time. The peak of the ΔF/F signal in response to 4-CmC exposure was used to assess SR Ca^2+^ content, as previously described [23].

### Calpain activity

Calpain activity was determined in whole EDL muscle homogenates (i.e., in the presence of its endogenous inhibitor calpastatin) using a calpain-specific fluorogenic substrate (*N*-succinyl-Leu-Tyr-7-amido-4-methylcoumarin, SLY-AMC), as previously described [25]. Briefly, 150 µg of muscle homogenates were incubated at 37°C in buffer solution (20 mM Tris–HCl, 5 mM CaCl_2_, 1 mM Dithiothreitol (DTT) 1, 0.15 nM Pefabloc and 5.8 nM Pepstatin A) for 10 min. After addition of 5 µl of 25 mM SLY-AMC in dimethyl sulfoxide, buffer was added to adjust the volume to 1 ml to obtain a SLY-AMC final concentration of 125 µM. The fluorescence signal of the released AMC was monitored using a Perkin Elmer spectrofluorimeter at 37°C for 15 min (excitation 380 nm, emission 460 nm). Control assays were performed without CaCl_2_ and in the presence of 10 mM EDTA and 10 mM EGTA. Calpain activity was determined as the Ca^2+^-dependent cleavage of SLY-AMC and was expressed as arbitrary units per minute of incubation per milligram of muscle protein.

### Immunoblotting

Muscle protein extracts were denatured in an equivalent volume of Laemmli buffer and incubated at 95°C for 4 min. Thirty mg of protein extracts were separated on a 6% SDS–PAGE column at a constant voltage of 150 V for 90 min. Proteins were transferred onto nitrocellulose membranes subjected to 210 mA of constant electric current for 2 h. Membranes were blocked with non-fat milk and incubated at 4°C with polyclonal anti-calpain 1 (Affinity Bio- Reagents, U.S.A.; dilution 1∶5000) and polyclonal anti-calpain 2 (previously described [26], dilution 1∶200) antibodies overnight. Specific interactions were detected with horseradish peroxidase conjugated secondary antibodies (Santa Cruz Biotechnology, Gaithersburg, U.S.A) and enhanced chemiluminescence solution (ECL Plus, Amersham, UK). Protein blots were quantified by scanning densitometry (Snapscan T1200, AGFA, France) with the Scion Image software. The calpain fractional activation index was calculated by dividing the band intensity of the autolysed form by the sum of the band intensities of both autolysed and non-autolysed isoforms.

### Statistical analysis

Values are expressed as the mean ± SEM. Statistical comparison of parameters in the two groups was carried out using a non-parametric Kruskal–Wallis test. Significance was set at p<0.05.

## Results

### Muscle morphometric and phenotypic changes following chronic treatment with clenbuterol

**Figure 1:**
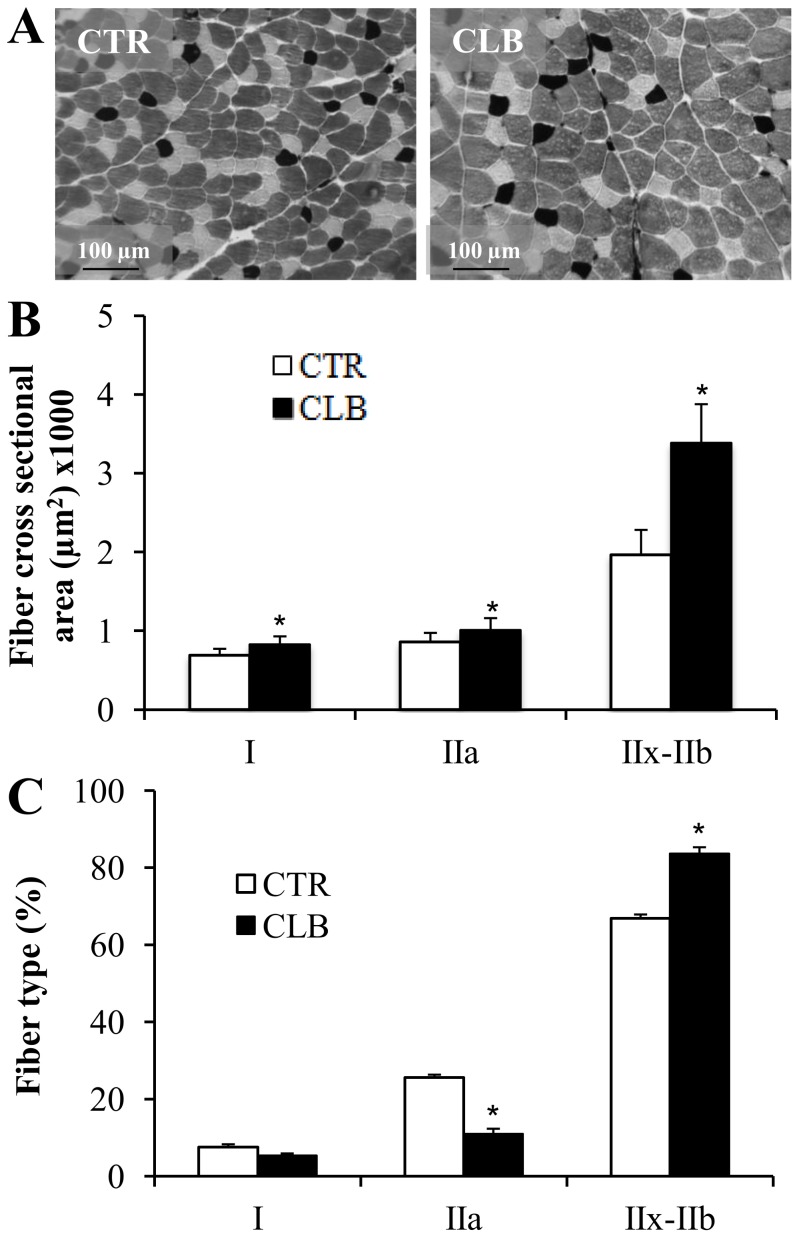
Effects of chronic clenbuterol treatment on EDL fiber-type profile and muscle-fiber cross-sectional area. **A,** Histochemical staining for Myosin ATPase in 6 µm EDL cryosections from control (CTR; untreated group) and clenbuterol-treated (CLB) animals. The images correspond to staining for ATPase activity. The dark stain highlights fibers that mainly express Myosin Heavy Chain-I (MyHC-I); the light stain indicates fibers that mainly express MyHC-IIa; and the intermediate color corresponds to fibers that mainly express MyHC-IIx-IIb. **B,** Quantification of Myosin ATPase staining revealed an increased cross-sectional area for all fiber types following clenbuterol treatment (In µm^2^, type I from 689.52±29.56 to 828.53±35.96, type IIa from 860.84±40.37 to 1009.43±54.18, type IIx-b from 1968.74±111.39 to 3390.55±173.64). * p<0.05 compared to CTR. **C,** Treatment with clenbuterol induced a phenotypic shift from slow-oxidative to fast-glycolytic profile (type I from xxx ± xx to xxx ± xx, type IIa from xxx ± xx to xxx ± xx, type IIx-b from xx ± xx to xxx ± xx). Values are expressed as the mean ± SEM (7 animals in each group). * p<0.05 compared to CTR.

To investigate the effect on muscle Ca^2+^ homeostasis of chronic clenbuterol treatment, 22 male Wistar rats received clenbuterol (CBL) or saline vehicle (CTL) for 21 days and then the EDL muscles were harvested for morphological and functional investigations. As previously described [1], chronic CBL administration resulted in a significant increase of the EDL muscle mass compared to controls (+22%; 220.9±5.4 mg CBL vs 176.3±8.3 CTL group; p<0.001; n = 11 rats/group). Muscle fiber types were identified based on the extent of myosin ATPase staining (Fig. 1A). As seen in Fig. 1B, CBL treatment led to an increase in the CSA of type I (+20%), type IIa (+17%) and type IIx-IIb (+72%) fibers. Fig. 1C shows that CBL treatment led to a shift from a slow-oxidative to a fast-glycolytic fiber type profile. While the percentage of type I fibers did not change significantly, a 17% decrease in type IIa fibers and a 15% increase in type IIx-IIb fibers were observed (p<0.05).

### Muscle functional features

**Figure 2:**
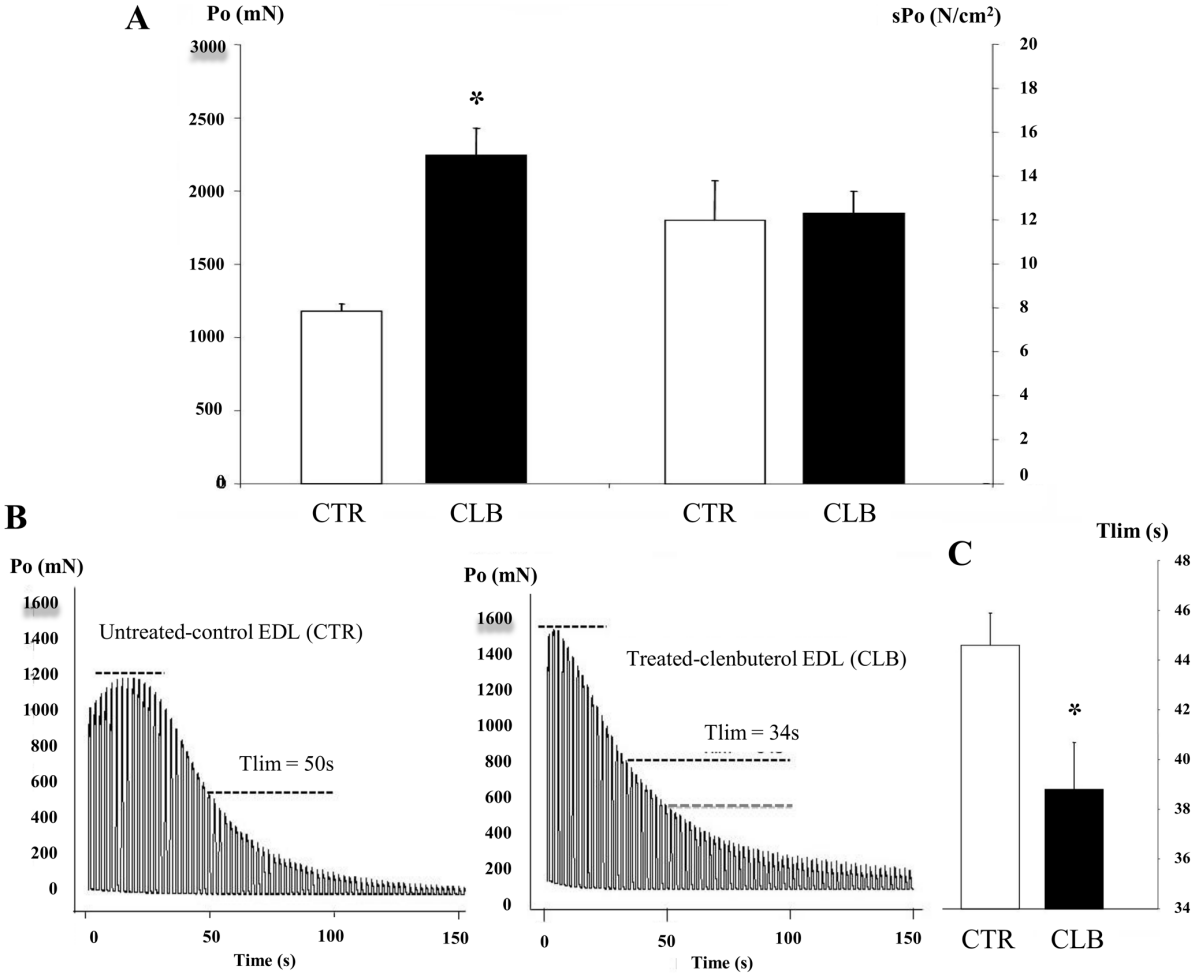
Effects of chronic clenbuterol treatment on intact muscle contractile properties. **A,** Effects of clenbuterol (CLB) treatment for 21 days on EDL maximal isometric tension. Left graph, P_0_: maximal isometric tetanic tension, in mN; Right graph, sP_0_ specific maximal isometric tetanic tension, in N/cm^2^. * p<0.05 compared to controls (CTR, untreated animals). **B,** Recordings show the decrease in tension resulting from the fatigue protocol in control EDL muscles (CTR) in which T_lim_ was almost 50 s and in clenbuterol treated EDL muscles (CLB) in which T_lim_ was about 34 s. Black dashed lines represent the maximal force and 50% of the initial force. The grey dashed line in the CLB panel represents 50% of the initial force of CTR for comparison. **C,** Mean ± SEM of the T_lim_ values/group (n = 15 for CTR; n = 22 for CLB). * p<0.05 compared to CTL.

Skeletal muscle mass is a key determinant of the force output. We thus investigated whether the EDL muscle hypertrophy and the shift to a fast-glycolytic muscle fiber type profile induced by CBL chronic treatment affected the force output. As expected, P_0_ was markedly increased after 21 days of CBL treatment (+87.5±2.3%) compared to CTL, whereas the sP_0_ values (i.e., normalization of the P_0_ values to the whole-muscle CSA), were not different between groups (Fig. 2A). Recording of the decline in tension in CTL and CBL-treated EDL muscles during the 5-min fatigue protocol (Fig. 2B) showed that the initial tension was higher and occurred earlier in CBL-treated EDL than in CTL muscles, in accordance with the increase in muscle mass and the phenotypic shift to a faster contractile profile observed in CBL-treated muscles. Conversely, the peak tetanic force decreased more quickly in CLB-treated muscles, and T_lim_, used as an index of resistance to fatigue, was significantly reduced compared to CTL. **C**BL treatment induced a mean 13±1% decrease in the T_lim_ (Fig. 2C; p<0.05), thus indicating that chronic treatment with CBL impairs muscle endurance.

### Ca^2+^-signaling is impaired by chronic treatment with clenbuterol

**Figure 3:**
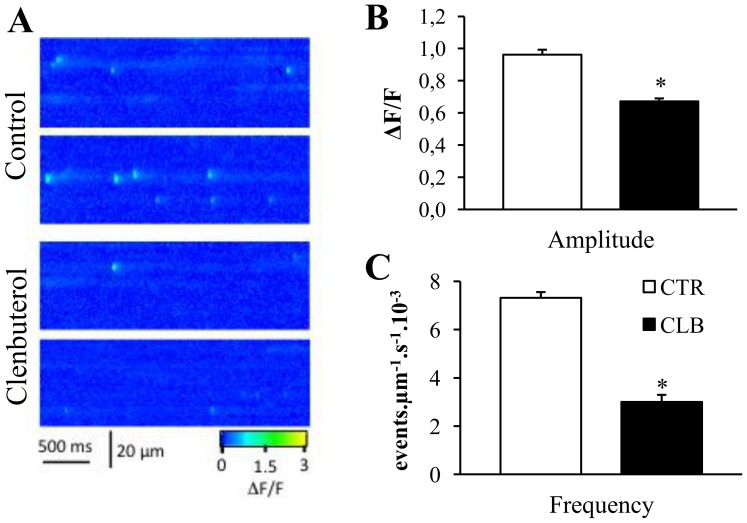
Effects of chronic clenbuterol treatment on Ca^2+^ spark amplitude and frequency. **A,** Representative line-scan Δ*F*/*F* images of permeabilized skeletal muscle fibers from control (top) and clenbuterol-treated rats (bottom). **B,** Comparison of Ca^2+^-spark amplitude in control (white bar graph) and clenbuterol-treated (black bar graph) animals. * p<0.05 compared to controls. **C,** Comparison of Ca^2+^-spark frequency in control (white bar graph) and clenbuterol-treated (black bar graph) animals. Values are expressed as the mean ± SEM of 10 animals/group. * p<0.05 compared to controls.

**Figure 4:**
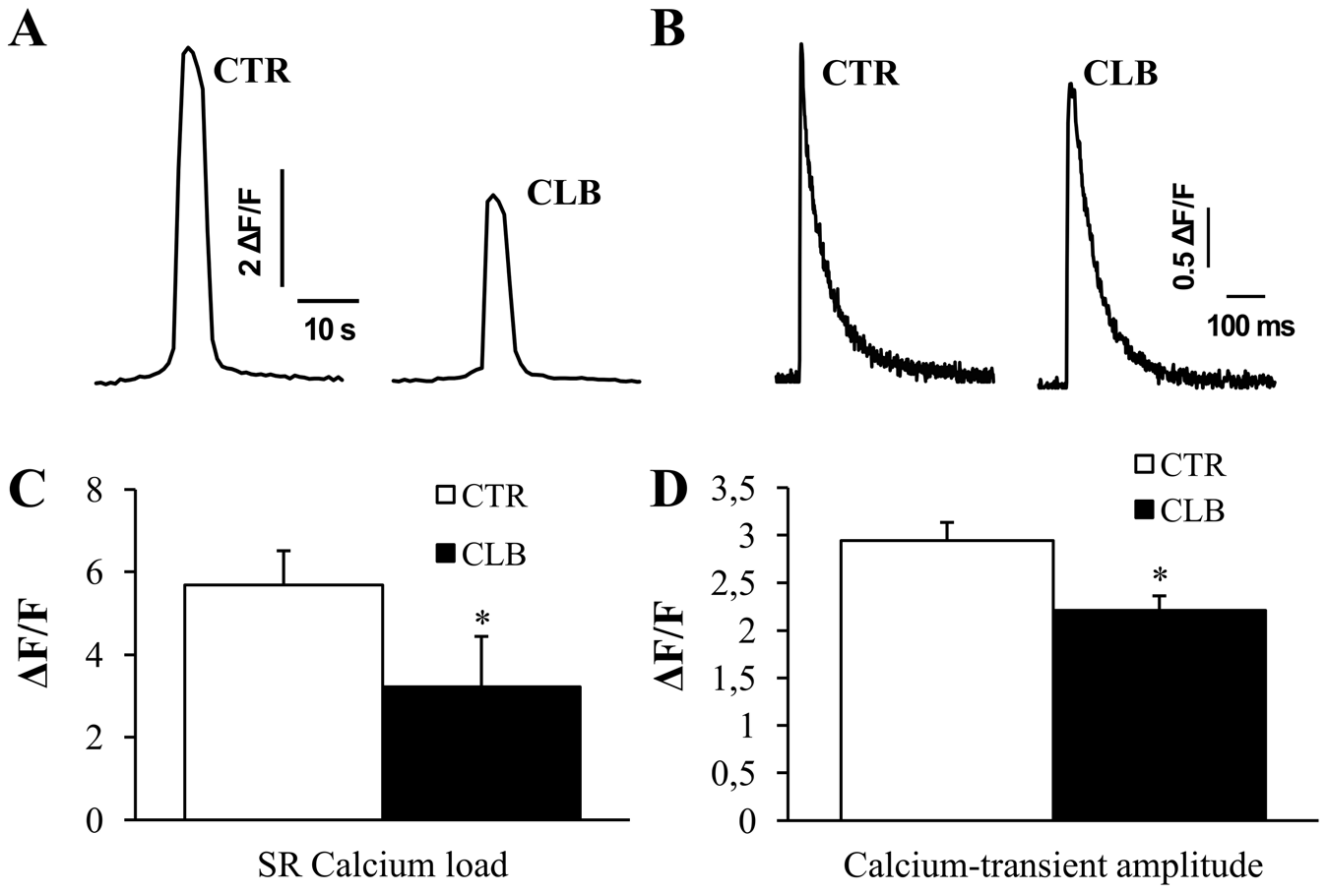
Effects of chronic clenbuterol treatment on SR Ca^2+^ load and Ca^2+^-transient amplitude. **A,** Typical representation of calcium release elicited with 50 µM 4-chloro-*m*-cresol in control (CTR) and clenbuterol-treated (CLB) animals. Ca^2+^-transient was imaged using fast time series of x–y confocal images. The amplitude (peak) of the signal was used as indicator of SR Ca^2+^ load. **B,** Typical representation of Ca^2+^-transient triggered by field stimulation with a single 2 ms pulse in intact EDL muscles loaded with 50 µM Rhod-2 AM from control (CTR) and clenbuterol-treated (CLB) animals. **C,** Comparison of SR Ca^2+^-load in control (CTR) and clenbuterol-treated (CLB) a nimals. Ca^2+^ release was elicited with 50 µM 4-chloro-m-cresol as shown in panel A. Values are expressed as the mean ± SEM of 10 animals in each group. * p<0.05 compared to controls. **D,** Quantification of Ca^2+^-transient amplitude in control (CTR) and clenbuterol-treated (CLB) animals. Ca^2+^-transients were triggered by field stimulation with a single 2 ms pulse as shown in panel B. Values are expressed as the mean ± SEM of 8 animals in each group. * p<0.05 compared to controls.

As Ca^2+^ signaling is a major regulator of skeletal muscle plasticity and functional properties, we evaluated whether CBL treatment could impair Ca^2+^ signaling and, more specifically, the frequency and spatio-temporal properties of spontaneous Ca^2+^ sparks, which reflect RyR gating properties. Spontaneous Ca^2+^ spark were monitored using a confocal system operated in line-scan mode (Fig. 3A). Analysis of their spatio-temporal properties indicated that the Ca^2+^ spark amplitude (Fig. 3B) and frequency (Fig. 3C) were drastically reduced in EDL muscles of rats treated with CBL (−30% and −59%, respectively, p<0.01 compared to controls), but their temporal properties were not significantly altered (rise time: 5.79±0.42 CBL vs 6.17±0.24 CTL; decay: 9.66±0.61 CBL vs 10.18±0.52 CTL). Assessment of SR Ca^2+^ load (peak of the signal, [23], [24]) using 4-CmC showed a 43% decrease (p<0.01) in 4-CmC-induced Ca^2+^ release in CBL-treated compared to CTL EDL muscles (peak of the signal, Fig. 4A), indicating that the SR Ca^2+^ content is reduced following chronic CBL treatment. In accordance with these results, the global Ca^2+^-transient amplitude was also reduced by 25% (p<0.01) after chronic CBL treatment compared to CTL (Fig. 4B).

### Calpain activity

**Figure 5:**
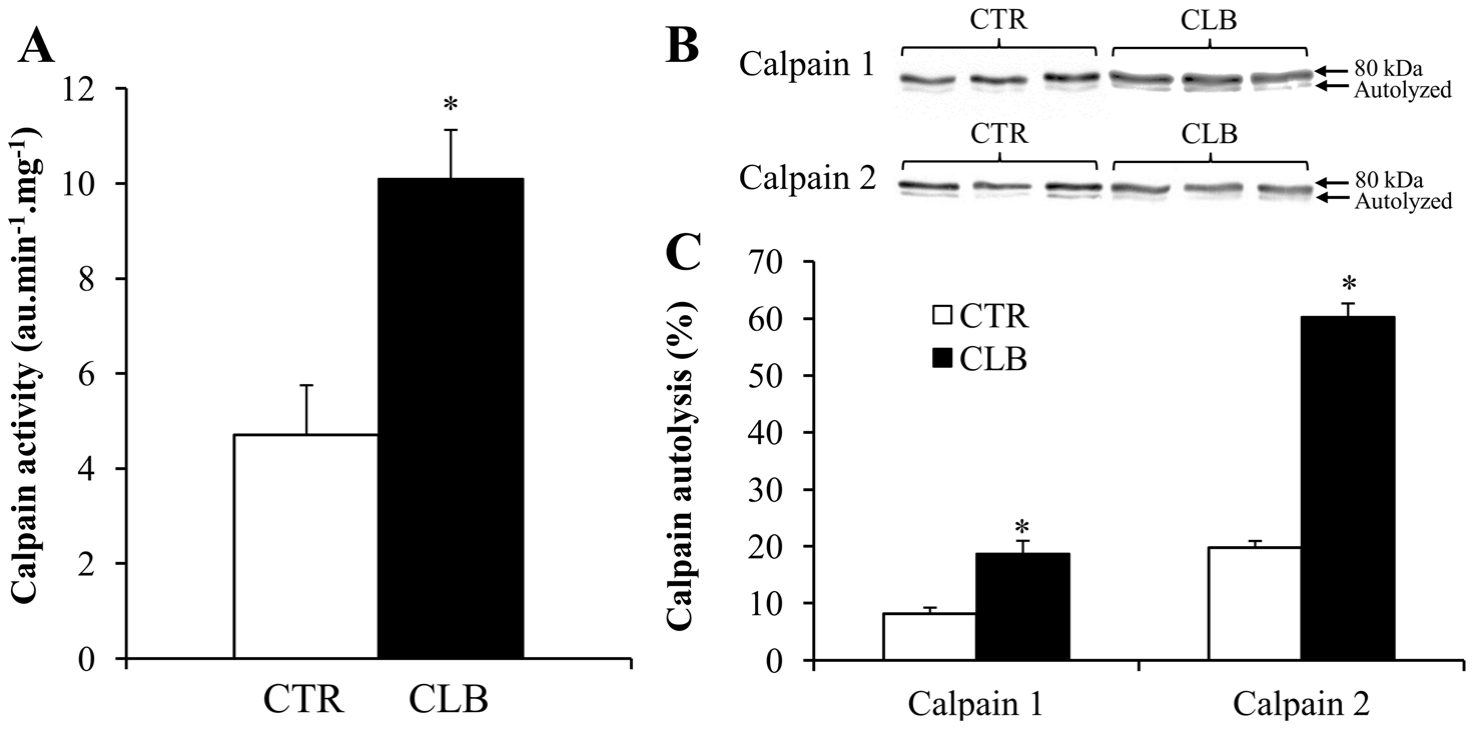
Effects of chronic clenbuterol treatment on EDL muscle calpain activity. **A,** Calpain activity was determined in whole EDL muscle homogenates using a calpain-specific fluorogenic substrate in control (CTR) and clenbuterol-treated (CLB) animals. * p<0.05 compared to controls. **B,** Western blot analysis with polyclonal anti-calpain 1 and polyclonal anti-calpain 2 antibodies of EDL muscle homogenates from control (CTR) and clenbuterol-treated (CLB) animals. **C,** Calculation of the calpain fractional activation index (i.e., percentage of autolysed calpains) in EDL muscle homogenates from control (CTR) and clenbuterol treated (CLB) animals. Values are the mean ± SEM of 8 animals in each group. * p<0.05 compared to controls.

Calpains could be involved in skeletal muscle remodeling induced by CBL and their activity is influenced by Ca^2+^ signaling (calpain 1 and 2 are also called μ- and m-calpain respectively, based on the µM or mM Ca^2+^ concentration needed for their activation). Global calpain activity, assessed by using spectrofluorimetric techniques, was drastically increased by 115% following CBL treatment (Fig. 5A, p<0.01 compared to CTL). This result was confirmed by immunoblotting experiments showing that autolysis of calpain 1 and calpain 2 was significantly increased in CBL-treated EDL muscle homogenates compared to CTL (from 8.14±3 to 18.73±5.94, p<0.01 for calpain 1; and from 19.74±3.41 to 60.34±6.64, p<0.01 for calpain 2) (Fig. 5B and C).

## Discussion

Clenbuterol induces muscle hypertrophy and a phenotypic shift to a faster contractile profile. These properties have made clenbuterol a promising candidate for the treatment of various myopathies. However, several side effects have been reported following chronic treatment with clenbuterol and their mechanisms have not been clearly elucidated yet. In this study, we demonstrate that a 21-day treatment with clenbuterol is associated with substantial impairment of Ca^2+^ signaling. In particular, we found that chronic clenbuterol treatment decreases SR Ca^2+^ load (Fig. 4A) and this could explain the altered SR Ca^2+^ release properties. In particular, we found that the amplitude and frequency of elementary Ca^2+^-release events (Ca^2+^-sparks; Fig. 3) and global electrically-evoked Ca^2+^-transients were reduced (Fig. 4B). We hypothesize that the decreased SR Ca^2+^ load after clenbuterol treatment may be due to a Ca^2+^ leak from RyR that could stimulate calpain activation and activity. Taken together, these alterations may contribute to the deleterious effects on muscle functionality reported following chronic treatment with clenbuterol.

### Effects of clenbuterol treatment on skeletal muscle mass and fiber-type profile

Because of its positive effect on muscle mass, clenbuterol therapeutic potential in various pathological conditions affecting muscle tissue has been extensively evaluated. Studies in animal models have demonstrated beneficial effects of treatment with β_2_-agonists in conditions of muscular dystrophy, disuse, aging, denervation or amyotrophic lateral sclerosis [27], [28], [29], [30], [31], [32].

Our 21-day treatment with clenbuterol led to marked EDL muscle hypertrophy (as indicated by muscle mass and muscle-fiber CSA measurements) and a phenotypic shift to a faster muscle profile. These effects are consistent with what is usually reported in the literature on fast skeletal muscle [3], thereby asserting the efficacy of our treatment.

### Effects of clenbuterol treatment on skeletal muscle function

Surprisingly, while the effects of β2-agonists on skeletal muscle mass and phenotype have been extensively studied, few experiments have evaluated the impact of chronic treatment with clenbuterol on skeletal muscle function. In humans, 4-week clenbuterol treatment in orthopedic patients did not improve muscle absolute strength [33]. In rats, two studies have demonstrated negative effects of chronic clenbuterol treatment on exercise performance evaluated with the run-to-exhaustion treadmill [34], swimming or sprinting protocol tests [35]. Increased skeletal muscle fatigability was also reported following clenbuterol treatment in rat [7], an effect associated with reduced skeletal muscle oxidative capacity [36], [37], [38]. Moreover, muscle hypertrophy following chronic clenbuterol treatment increases the isometric force in fast-twitch skeletal muscle [7]. Our study confirms these previous results. The absolute force (P_0_) of EDL muscle was increased following clenbuterol treatment, while the specific force (sP_0_) was not different compared to controls, suggesting that the strength improvement was only due to the increased skeletal muscle mass. Analysis of representative recording of the decrease in EDL tension during the fatigue protocol highlighted that the initial tension was higher and appeared earlier in clenbuterol-treated EDL muscles than in untreated controls, in accordance with the increase in muscle mass and the phenotypic shift to a faster contractile profile. Fatigability of EDL muscles from clenbuterol-treated animals was nevertheless increased, as the peak tetanic force fell more quickly in clenbuterol-treated EDL muscles than in controls, and T_lim_, used as an index of resistance to fatigue, was significantly reduced. This last result confirmed that clenbuterol treatment negatively affects muscle endurance, in agreement with the phenotypic remodeling.

### Ca^2+^ signaling impairment induced by clenbuterol treatment: a potential explanation for the impaired skeletal muscle function?

Ca^2+^ signaling plays a critical role in regulating skeletal muscle functional properties [13]. Different studies have demonstrated that acute application of clenbuterol on skeletal muscle fibers profoundly affects Ca^2+^ homeostasis, resulting in an increased Ca^2+^ leak from the SR. To date, no studies have evaluated the impact of chronic treatment with clenbuterol on skeletal muscle Ca^2+^ signaling. It is generally accepted that Ca^2+^ spark spatio-temporal properties and frequency reflect RyR1 function, as indicated by previous publications by our group. For instance, we previously used Ca^2+^ spark analysis to demonstrate the contribution of RyR in skeletal muscle pathophysiology [19], [22]. Here, we found that the frequency and amplitude of spontaneous elementary SR Ca^2+^-release events (Ca^2+^-sparks) are reduced in EDL muscle fibers from clenbuterol-treated animals compared to control, suggesting changes in the SR Ca2+ release properties. The decreased amplitude of Ca^2+^ sparks could be explained by a decreased SR Ca^2+^ load as suggested by results in cardiac myocytes [39]. The presence of Ca^2+^ regulatory sites on the luminal side and on the myoplasmic side of the SR could explain how a reduction in SR Ca^2+^ content might affect Ca^2+^ release [40]. Indeed, it has been already shown that experimental decrease in SR Ca^2+^ load reduces Ca^2+^-spark frequency and amplitude in smooth muscle from rat [41]. In agreement with these results, we also found that in EDL muscles from clenbuterol-treated animals SR Ca^2+^ load was markedly reduced compared to untreated controls. The decrease in SR Ca2+ load could be a consequence of increased passive Ca2+ leak from the SR as already reported following acute clenbuterol treatment in mechanically skinned skeletal muscle fibers from rats [14]. We also observed a decrease in the amplitude of global Ca^2+^-transients in EDL muscles from clenbuterol-treated animals. This result is in accordance with the reduced amplitude of Ca2+ sparks and may also be a consequence of the reduced SR Ca2+ load. A reduction in Ca^2+^-transient amplitude could induce a decrease in the maximum capacity of force production by skeletal muscle, as already demonstrated following acute clenbuterol treatment [14]. However, we did not find any deterioration of the specific maximal isometric strength in EDL muscles following chronic treatment. An explanation of this apparent discrepancy could be a compensatory mechanism at the level of the myofilament Ca^2+^ sensitivity. Indeed, it has already been reported that chronic clenbuterol administration can improve Ca^2+^ sensitivity in rat slow-twitch skeletal muscle fibers [9].

### Clenbuterol treatment increases calpain activities: a consequence of disturbed calcium homeostasis?

Ca^2+^ is a second messenger that regulates many signaling pathways. Therefore, Ca^2+^ homeostasis disturbances may also potentially disturb skeletal muscle metabolic and catabolic processes. For example, Ca^2+^ participates in the regulation of calpain activity. In particular, calpain 1 (μ-calpain) binds Ca^2+^ rapidly when the calcium concentration increases within a physiological range [42]. The role of calpain 2 (m-calpain) in the regulation of skeletal muscle function *in vivo* is more ambiguous, due to its apparent requirement of supraphysiological Ca^2+^ concentrations [43]. We hypothesized that a Ca^2+^ leak from the SR could mediate a decreased SR Ca^2+^ load and finally may reduce Ca^2+^-sparks and global Ca^2+^-transient amplitude. Another consequence of the increased Ca^2+^ leak from the SR could be the activation of calpains. Such association between SR Ca^2+^ release disturbances and calpain activation has already been reported in a mouse model of Duchenne muscular dystrophy (mdx mouse), a pathological condition that is associated with increased SR Ca^2+^ leak [46], reduced amplitude of Ca^2+^-transients [45] and increased calpains activity [44]. This example reinforces our hypothesis on a potential link between SR Ca^2+^ regulation disturbances and calpain activation. Recent studies have confirmed that chronic clenbuterol treatment in rats leads to increased calpain activities in fast-twitch skeletal muscles [11], [47]. Previously, it has been reported that clenbuterol treatment could decrease calpain 1 activity and increase calpain 2 activity [47]. Here, we found that chronic treatment with clenbuterol increases both calpain 1 and 2 activities in EDL muscles. While calpain activation seems to be necessary for clenbuterol-induced hypertrophy and the fiber profile shift [48], its activation might also cause deleterious effects on skeletal muscle, for example by promoting fiber necrosis, as already suggested [42]. Further studies are needed to elucidate more precisely the consequences of increased calpain activities and the link with disturbed Ca^2+^ homeostasis. In particular, it would be interesting to evaluate the potential role of calpain activation in SR Ca^2+^ release changes. Indeed, Ca^2+^-activated proteases can induce proteolysis of SR Ca^2+^ release channels [49]. Moreover, in toad muscle fibers, Ca^2+^ exposure results in disruption of excitation-contraction coupling (E-C coupling) and titin, an effect possibly mediated, at least in part, by Ca^2+^-activated proteases [50]. Similar results have been reported in mammalian skeletal muscle fibers in which calpain 1 was considered a good candidate to explain the Ca^2+^-induced disruption of E-C coupling [51], potentially mediated by junctophilin proteolysis [52]. Based on these studies, we could hypothesize that increased calpain activity induced by clenbuterol treatment might contribute to the disruption of E-C coupling and then SR Ca^2+^-release, leading to a functional impairment of skeletal muscle.

### Conclusion

In this study we demonstrate that chronic clenbuterol treatment induces significant changes in various Ca^2+^ signals associated with excitation-contraction coupling in fast-twitch skeletal muscles. This effect occurs in association with increased calpain activities. Together, these alterations might contribute to the structural and functional adaptations of skeletal muscle to chronic clenbuterol treatment. As clenbuterol is considered a good candidate for the treatment of muscle disuse, our study underlines that more experiments are necessary to further elucidate the impact of chronic clenbuterol treatment on muscle metabolism and function.
